# Effects of infant bronchiolitis on family life

**DOI:** 10.3389/fped.2024.1343045

**Published:** 2024-06-19

**Authors:** Rémy Assathiany, Marc Sznajder, Fabienne Cahn-Sellem, Claire Dolard, Andreas Werner

**Affiliations:** ^1^ Association Française de Pédiatrie Ambulatoire (AFPA), Ancenis, France; ^2^ MPedia, Bordeaux, France

**Keywords:** bronchiolitis, RSV, epidemic, family, family life, familial impact

## Abstract

**Background:**

Bronchiolitis is a respiratory infection of viral origin and is often linked to syncytial respiratory virus. It is the most frequent cause of hospitalisation in children aged under 2 years and sometimes requires transfer to intensive care. Infectious complications may also arise in the short term, and longer-term progression towards asthma is also possible. The occurrence of bronchiolitis in children may affect families in different ways, and may have psychological, organisational, employment-related, and possibly financial consequences.

**Objective:**

The aim of the study was to determine the familial and socioeconomic repercussions of bronchiolitis in infants.

**Setting:**

Parents with a child with bronchiolitis between January 2021 and May 2022, who were registered at the site of the Association Française de Pédiatrie Ambulatoire (Mpedia site) or at the site of the Malin Programme, which serves families experiencing financial difficulties, were included in the study.

**Participants:**

All parents consenting to participate in the study.

**Results:**

A total of 2,059 valid questionnaires were retrieved: 1,318 (64%) were obtained from parents through the Mpedia website and 741 (36%) were obtained through the Malin Programme. Parents associated with the Malin Programme had more children, as well as higher rates of unemployment and financial difficulties, and required greater medical assistance. Hospitalisation was necessary in 37% of cases and was comparable between groups. During the illness, moderate to severe anxiety was present in 73% of parents; this percentage rose to 87% if the child required hospitalisation. Many parents reported effects on daily home (84%) and work life (90%), and 60% had taken a leave of absence from work.

**Conclusion:**

Beyond the immediate or longer-term medical consequences of bronchiolitis in infants, the illness affects families in multiple ways and can lead to anxiety, as well as changes in day-to-day home and work life. Physicians should have greater awareness of these consequences and should strive to decrease their impact.

## Introduction

Bronchiolitis, a viral illness that leads to obstruction of the bronchioles, infects infants aged under 2 years during epidemics that occur during the fall and winter months. Respiratory syncytial virus (RSV) is the virus most often responsible for this condition. In an annual cohort, the number of infants affected by bronchiolitis has been estimated to be 20%, thus indicating that it is the major cause of hospitalisation in infants aged under 1 year in developed countries. Each year, 2%–3% of infants, particularly younger infants, are hospitalised for bronchiolitis (1) for an average of 5.1 days (2). Transfer to intensive care, mainly for oxygen administration, is required for 1%–2% of hospitalised infants (3). Mortality rates associated with bronchiolitis are high in developing countries but are almost nil in North America and Europe (4). Literature data examining trends in the numbers of infants hospitalised for bronchiolitis and the number transferred to intensive care are conflicting. In England, the number of infants hospitalised for bronchiolitis grew by a factor of 7 between 1979 and 2011 (5), whereas this number was stable in Ontario between 2004 and 2018 (6), and even decreased in the United States between 2000 and 2016 (7). Those studies have also reported that the number of infants transferred to intensive care was either stable (5) or increased (6, 7). In Europe, the number of cases has also increased in the past 20 years (8). In France, during the winter of 2020–2021, 39% of children presenting with bronchiolitis in a hospital emergency department were hospitalised (9).

The progression of this very common illness is marked by immediate or longer-term medical complications. Among immediate complications, superinfection should be noted, as well as acute middle-ear infection, which occurs in 57% of children aged under 3 years (10). Acute pyelonephritis is much less common, but the possibility of its occurrence should be systematically examined in infants with high fever (11). Among later-onset complications, infants who contract bronchiolitis in the first 6 months of life have an elevated probability of occurrence of acute middle-ear infection, pneumonia, and infections requiring antibiotic treatment between the ages of 6 and 12 months (12). Later development of asthma is frequent, particularly if a rhinovirus is responsible for the bronchiolitis (13); the mechanism, which remains under discussion, appears to involve environmental or genetic factors (14), among which individual and/or familial atopic tendencies appear to play a determining role (15).

Beyond these medical complications, hospitalisation of an infant is a stressful event for any family and can have profound consequences for both parents and siblings. These consequences may be psychological in nature, arising from the stress of hospitalisation, or may involve changes in the family's employment, financial, and daily life situations. These non-medical effects of bronchiolitis are scarcely documented.

The aim of the present study was to evaluate the familial and socioeconomic consequences of the occurrence of bronchiolitis within family units.

## Materials and methods

We did not use the questionnaire from a prior study, because it was complex and pertained to only hospitalised infants (16). Instead, we developed a questionnaire consisting of closed-ended questions, which was validated by the Association Française de Pédiatrie Ambulatoire (AFPA) committee of experts. The questionnaire included questions regarding how the illness was managed, as well as its psychological, organisational, and work-related consequences on the families. Information relating to family socioeconomic status was also requested.

The criterion for inclusion was the occurrence of bronchiolitis in an infant aged under 2 years between January 2021 and the date of administration of the questionnaire (April–May 2022). The data were collected using SurveyMonkey® software (SurveyMonkey Inc.).

The anonymous questionnaire was administered in April and May 2022 to 9,300 parent's registered with the parent website of the AFPA (www.mpedia.fr). The questionnaire was also made available on social networks (i.e., Facebook and Instagram). To counterbalance the over-representation of higher socioeconomic categories among families registered at the AFPA website, the questionnaire was also distributed to all 47,146 families registered with the Malin Programme. The Malin Programme is a non-profit recognised association within the scope of the French law of 1901; it aims to give parents, particularly those under tight budgetary constraints, advice from pregnancy onwards; this advice comes from French paediatric health societies and concerns nutrition, breastfeeding, sleep, screens, and physical activity. All families can register on the website (programme malin.fr); financial assistance may be granted for families in precarious situations, which are defined by the agency responsible for child allowances according to the ratio of family income to family size.

Parents who completed the questionnaire on the Mpedia website were denoted Mpedia families or parents, whereas those belonging to the Malin Programme were denoted Malin Programme families or parents. All questionnaires, including those with partial responses, were considered.

The study was approved by the AFPA ethics committee.

### Statistical analyses

The only data analysed were categorical in nature and are presented as frequencies and proportions. The results were compared between groups (Mpedia vs. Malin Programme) using Fisher's exact test. The level of significance was set to 0.05. Statistical analyses were conducted using GraphPad QuickCalcs software.

## Results

A total of 2,059 questionnaires were partially or fully valid: 1,318 (64%) were obtained from the parents on the Mpedia website and 741 (36%) were obtained from parents participating in the Malin Programme. Responses were obtained from all French departments except five (Creuse, Gers, Lozère, Nièvre, and Yonne), and the largest number of responses was received from the Gironde. A large proportion of questionnaires was completed by mothers (94%) aged over 25 years (95%).

### Family characteristics

Table 1 presents the characteristics of the families from the two groups. Parents from the Malin Programme were characterised by younger maternal age, less frequent rates of cohabitation as couples (83% vs. 99%; *p* *< *0.0001), and more children. Moreover, higher rates of unemployment were observed among these parents (58% vs. 13%; *p* < 0.0001). In terms of employment categories, parents in the Mpedia group were mainly managers and professionals (43% vs. 6%; *p* < 0.0001). Families who were part of the Malin Programme had higher rates of financial difficulty (47% vs. 10%; *p* < 0.0001) and had greater recourse to medical assistance programs (33% vs. 3%; *p* < 0.0001) such as Couverture Maladie Universelle (CMU) or Aide Médicale de l'Etat (AME).

**Table 1:** Family characteristics.

	Total	Mpedia	Programme Malin	*p*
Person responding	*N* = 2,059	*N* = 1,318	*N* = 741	
Mother	1,944 (94%)	1,262 (96%)	682 (92%)	0.0006
Father	115 (6%)	56 (4%)	59 (8%)	
Mother's age (years)	*N* = 2,054	*N* = 1,316	*N* = 738	
<25	109 (5%)	27 (2%)	82 (11%)	<0.0001
>25	1,945 (95%	1,289 (98%)	656 (89%)	
Living arrangement	*N* = 1,735	*N* = 1,149	*N* = 586	
As a couple	1,620 (93%)	1,135 (99%)	485 (83%)	<0.0001
Isolated single parent	115 (7%)	14 (1%)	101 (17%)	
Number of children	*N* = 1,734	*N* = 1,148	*N* = 586	
1	624 (36%)	499 (43%)	126 (21%)	<0.0001
>1	1,110 (64%)	649 (57%	460 (79%)	
Premature birth	*N* = 1,993	*N* = 1,285	*N* = 708	
	218 (11%)	130 (10%)	88 (12%)	0.03 (NS)
Living environment	*N* = 1,730	*N* = 1,144	*N* = 586	
Urban	1,082 (63%)	719 (63%)	363 (62%)	0.7 (NS)
Rural	648 (37%)	425 (37%)	223 (38%)	
Occupation	*N* = 1,747	*N* = 1,150	*N* = 597	
Managerial/professional	524 (30%)	489 (43%)	35 (6%)	<0.0001
Intermediate profession	193 (11%)	168 (15%)	25 (4%)	
Salaried employee	410 (23%)	292 (25%)	118 (20%)	
Wage worker	47 (3%)	17 (1%)	30 (5%)	
Others	81 (5%)	40 (3%)	41 (7%)	
Unemployed	492 (28%)	144 (13%)	348 (58%)	
Financial status	*N* = 1,734	*N* = 1,149	*N* = 585	
Comfortable/stable	1,339 (77%)	1,031 (90%)	308 (53%)	<0.0001
Precarious	395 (23%)	118 (10%)	277 (47%)	
Medical assistance	*N* = 1,747	*N* = 1,150	*N* = 597	
CMU or AME	240	40 (3%)	200 (33%)	<0.0001

*N*, number of responses to the question; NS, not significant.

### Management of bronchiolitis

Because of missing or incomplete data, 1,993 files concerning the management of bronchiolitis were usable. Table 2 details how the occurrence of bronchiolitis was managed. The number of medical consultations varied, and 58% of the infants had two or more consultations. Hospitalisation was necessary in 37% of cases, typically for more than 3 days; this percentage was comparable between groups. In total, 75 (10%) infants required a transfer to another hospital, most often in another city. The distribution of transfers in the same city versus another city was significantly different in the two groups: they were more prevalent among Malin Programme families (4% vs. 0%; *p* *< *0.0048). Transfer to intensive care was required in 144 cases, which amounted to 7% of the total number of bronchiolitis cases studied.

**Table 2:** Management of bronchiolitis.

	Total	Mpedia	Programme Malin	*p*
Number of responses	1,993	1,284	709	
Number of consultations				
0	107 (5%)	57 (4%)	50 (7%)	0.06 (NS)
1	731 (37%)	501 (39%)	230 (32%)	
2	622 (31%)	427 (33%)	195 (28%)	
>2	533 (27%)	299 (23%)	234 (33%)	
Hospitalisation	731 (37%)	455 (35%)	276 (39%)	0.13 (NS)
Length of hospitalisation (days)				
1–3	247 (34%)	140 (31%)	107 (39%)	0.036
>3	476 (65%)	308 (69%)	168 (61%)	
Hospital transfer				
Same city	14 (2%)	1 (0%)	12 (4%)	0.0048
Different city	61 (8%)	31 (7%)	30 (11%)	
Stay in intensive care	144 (7%^a^)	87 (7%^a^)	57 (8%^a^)	0.6 (NS)

NS, not significant.

^a^ Percentage on total number of bronchiolitis.

### Consequences of illness among families

The consequences of a child's illness on the family were manifold and varied (Table 3). Moderate to severe anxiety was reported by 73% of parents after the initial diagnosis, and higher rates of anxiety were reported as the illness progressed (82%), particularly in cases requiring hospitalisation (87%). The effects on daily life were considerable and were reported by 84% of parents. Changes in childcare arrangements were reported by 20% of families, more often in the Mpedia group (23% vs. 15%; *p* = 0.0004).

**Table 3:** Familial impact of bronchiolitis.

	Total	Mpedia	Programme Malin	*p*
Number of responses	1,841	1,206	635	
Change in childcare arrangements	374 (20%)	274 (23%)	100 (15%)	0.0004
Impact on daily life	1,539 (84%)	1,060 (88%)	479 (75%)	<0.0001
Anxiety upon diagnosis				
Absent or slight	454 (25%)	306 (25%)	148 (24%)	0.73 (NS)
Moderate or severe	1,343 (73%)	893 (74%)	450 (72%)	
Anxiety during the illness				
Absent or slight	285 (15%)	176 (15%)	109 (17%)	0.065 (NS)
Moderate or severe	1,516 (82%)	1,023 (85%)	493 (77%)	
Anxiety during hospitalisation				
Number of responses	731	445	286	<0.0001
Absent or slight	92 (13%)	39 (9%)	53 (19%)	
Moderate or severe	639 (87%)	406 (91%)	233 (81%)	

NS, not significant.

### Employment-related consequences of illness

Approximately 90% of parents reported that the child's illness had affected the quality of their work (Table 4). Almost two-thirds (60%) of parents took a leave of absence because of the child's illness, and 13% were absent from work for more than 5 days.

**Table 4:** Impact on work during bronchiolitis.

	Total	Mpedia	Programme Malin	*p*
Employment situation	*N* = 1,963	*N* = 1,273	*N* = 690	
Actively employed	929 (47%)	754 (59%)	175 (25%)	<0.0001
Unemployed	1,034 (53%)	519 (41%)	515 (75%)	
	*N* = 929	*N* = 754	*N* = 175	
Impact on work	823 (88%)	648 (86%)	175 (100%)	<0.0001
Time off work	566 (60%)	462 (61%)	104 (59%)	0.71 NS
Length of time off work (days)	*N* = 459	*N* = 381	*N* = 78	
≤5	387 (84%)	325 (85%)	62 (80%)	0.23 (NS)
>5	72 (16%)	56 (15%)	16 (20%)	

*N*, number of responses to the question; NS, not significant.

## Discussion

This study involving more than 2,000 families allowed us to evaluate the effects of the occurrence of bronchiolitis on families. In this study, the questionnaires were completed primarily by mothers, as previously reported (17). A total of 37% of infants with bronchiolitis were hospitalised, findings comparable to those of *Santé Publique France*, in which 39% of such infants were hospitalised after presenting to the emergency department in the 2020–2021 winter (9). The hospitalisation rate was comparable in the two sub-populations studied, whereas in the United States, it was higher in families in a precarious socio-economic situation (18, 19).

The non-medical consequences observed in families were manifold and varied, and affected not only family life and organisation, but also parental work life. Anxiety is reported by three-quarters of parents responding to the survey; such anxiety when confronted with an illness involving respiratory distress, is legitimate and understandable; it would be even stronger in the case of bronchiolitis than in the case of infant hospitalisation for other reasons (20); this is understandable, as seeing his child struggling to breathe is very stressful for a parent. In our study, anxiety is more important in the Mpedia parents; indeed, it seems that the most qualified employees and managers are most at risk for anxiety symptoms (21); a previous study of parents of children hospitalized for bronchiolitis has also shown that this type of anxiety is proportional to the parents' number of years of education (17). Paradoxically, in this last study, higher levels of anxiety were not observed in cases in which the hospitalised infants had been born prematurely or experienced congenital cardiopathy, although these conditions are risk factors for hospitalisation in bronchiolitis (17). Good relationships with caregivers whom parents can trust is an important factor in relieving anxiety in this situation (22). Even if parents' anxiety attenuates over time, they may continue to experience difficulties several months after hospitalisation; thus, quality of life can be diminished for as many as 9 months after the occurrence of bronchiolitis (17, 20), particularly if atopic dermatitis is associated (23).

In our study, taking a leave of absence from work, which enabled parents to be present at the sick child's bedside, was reported by 60% of the families participating in the questionnaire. In the Malin Programme study sample, only 25% of parents were employed when the child was diagnosed with bronchiolitis, and all reported that the illness had affected their work.

The frequency at which parents took time off work for a child's bronchiolitis (60%) was comparable to findings from a Finnish study reporting that 52% of parents were absent from work for an average of 2.6 days; notably, taking time off work was more prevalent among parents of the youngest children (10). Another research study focusing on Spain and Italy has reported that 60% of parents took more than 1 week off work (22). Moreover, a study in Canada has observed that both parents spent an equivalent of 7 days at the hospital, thus amounting to a 3-day absence from work for each parent (2).

It is probably not easy to focus on your work when your child is hospitalised; this may affect the quality of the work performed. Thus, almost all parents (88%) who continued their daily activities during the child's illness indicated that the quality of their work had declined; a comparable frequency (82%) has been found in a Canadian study (2). Moderate to severe effects on work quality have also been reported by 37% of parents in a European study (22).

These complications must be considered by medical and paramedical personnel to help families successfully navigate this period of anxiety, which also affects home and work lives.

The present study has some limitations. First, we performed a retrospective analysis in which parents were questioned about the child's illness, which could have occurred as many as 17 months earlier. This aspect of the design could have led to recollection bias, although the occurrence of bronchiolitis in an infant is certainly a major event for any family. Moreover, additional statistical analyses, such as regression analyses, were not performed for this study; further analysis is needed in future studies to determine the true associations between the socioeconomic status of a family and the familial impacts of bronchiolitis.

The high occurrence rate of bronchiolitis, which affects 30% of infants each winter (8), justifies the development of a preventive vaccine to curb more severe forms of bronchiolitis and limit transmission. A vaccine could also decrease medical and non-medical complications of bronchiolitis, as well as the number of antibiotic prescriptions, as have been observed after the development of the influenza vaccine (24). RSV infection was previously preventable only in severely premature babies and certain other at-risk children (e.g., those with severe cardiopathy or immunodeficiency) by administering monoclonal antibodies (palivizumab), but administration had to be repeated every month during the period of community RSV infection risk. In 2020, 38 candidate vaccines were under development by the pharmaceutical industry, 19 of which were undergoing clinical trials (25). Two recent studies have shown encouraging results. The first involves a vaccine administered to pregnant women, which provides early protection for infants, and has shown good tolerance and satisfactory transplacental passage of protective antibodies (26). The second uses a long-lasting monoclonal antibody (nirsevimab) that can be injected once into an infant and has been demonstrated to effectively prevent RSV infections (27). This vaccine was certified by the European Commission in November 2022 and has been available in France since September 2023 to all infants aged under 1 year.

## Conclusion

This study, based on a sample of more than 2,000 families, highlights the importance of the psychological, familial, and socioeconomic effects of infant RSV infections on families. These consequences may sometimes be minimised or misunderstood by caregivers. Although these consequences were observed in both sub-populations studied, their importance varied. In the Mpedia group, anxiety during the illness—particularly during hospitalisation if required—was more frequent, whereas parents in the Malin Programme group reported more profound effects on work life. A complementary sociological study could define these differences more precisely and deepen understanding of this issue.

## Data Availability

The raw data supporting the conclusions of this article will be made available by the authors, without undue reservation.
